# Candy box technique for the fixation of inferior pole patellar fractures: finite element analysis and biomechanical experiments

**DOI:** 10.1186/s12891-023-06946-1

**Published:** 2023-10-23

**Authors:** Wei Fan, Jinhui Liu, Xiaoqi Tan, Daiqing Wei, Yunkang Yang, Feifan Xiang

**Affiliations:** 1 Department of Orthopaedics, The Affiliated Hospital of Southwest Medical University, Luzhou, China; 2 Sichuan Provincial Laboratory of Orthopaedic Engineering, Luzhou, China; 3 Department of Dermatology, Affiliated Hospital of Southwest Medical University, Luzhou, China

**Keywords:** Finite element analysis, Knee, Biomechanical experiments, Inferior Pole of the patella, Fracture, Candy box technique

## Abstract

**Background:**

Maintaining effective reduction and firm fixation in inferior pole patellar fractures is a highly challenging task. There are various treatment methods available; although tension-band wiring combined with cerclage wiring (TBWC) is the mainstream approach, its effectiveness is limited. Herein, we propose and evaluate a new technique called candy box (CB), based on separate vertical wiring (SVW), for the treatment of inferior pole patellar fractures. Specifically, we provide biomechanical evidence for its clinical application.

**Methods:**

Five fixation models were built: SVW combined with cerclage wiring (SVWC); TBWC; modified SVW with the middle (MSVW-A) or upper (MSVW-B) 1/3 of the steel wire reserved, and CB. A finite element analysis was performed to compare the displacement and stress under 100-N, 200-N, 300-N, 400-N and 500-N force loads. Three-dimensional printing technology was utilized to create fracture models, and the average displacement of each model group was compared under a 500-N force.

**Results:**

The results of the finite element analysis indicate that CB technology exhibits significantly lower maximum displacement, bone stress, and wire stress compared to that with other technologies under different loads. Additionally, in biomechanical experiments, the average force displacement in the CB group was significantly smaller than that with other methods under a 500-N force (P < 0.05).

**Conclusions:**

CB technology has the potential to overcome the limitations of current techniques due to its superior biomechanical characteristics. By incorporating early functional exercise and ensuring strong internal fixation, patient prognosis could be enhanced. However, further clinical trials are needed to fully evaluate the therapeutic effects of CB technology.

**Supplementary Information:**

The online version contains supplementary material available at 10.1186/s12891-023-06946-1.

## Background

The inferior pole of the patella refers to the cancellous bone in the portion of the lower 1/4 of the patella that is not covered by articular cartilage [1, 2]. Fractures of the inferior pole of the patella are thus extra-articular fractures and account for approximately 9.3–22.4% of patellar fractures [3–6]. Inferior pole patellar fractures are notorious; owing to the small fracture block and concentration of stress, most are comminuted fractures, and it is difficult to maintain effective reduction and firm fixation [7]. The patella is an important component of the knee extensor system, and inferior pole patellar fractures disrupt the integrity and continuity of this system, as well as the normal anatomical and biomechanical relationships of the patellofemoral joint [8]. Therefore, the correct treatment of these fractures is essential for restoring stability, integrity, and function of the knee extensor system [1, 9].

However, currently, there is no established standardized treatment for inferior pole fractures of the patella [10, 11]. The use of inferior pole excision surgery has been gradually abandoned [12]. Classic tension-band wiring combined with cerclage wiring (TBWC) has been used for a long time in the treatment of patellar fractures [13]; it is simple to perform and cost-effective, and is considered as the gold standard for internal fixation of patellar fractures. However, it is difficult to control separation and displacement during knee flexion for distal patellar fractures [14]. The basket plates technique is effective in reducing complications caused by TBWC, such as separation and displacement, achieved by applying pressure in multiple directions. However, this method does not guarantee early functional exercise of the knee joint and carries a potential risk of patellar tendon injury. [15, 16]. Further, the patellar claw technique provides a more suitable biomechanical environment for fixing and healing transverse fractures of the patella [17]. However, for inferior pole fractures of the patella, the excessive rebound force with this technique may pose risks such as re-displacement of small bone fragments [18]. The separate vertical wiring (SVW) technique was recently proposed as a new treatment method for inferior pole patellar fractures [19, 20], and some researchers have used SVW combined with cerclage wiring (SVWC) to treat inferior pole patellar fractures in patients with osteoporosis, achieving good results. However, this technique still does not allow early weight-bearing or full extension exercises and carries the risk of internal fixation failure [21]. In a 2018 report [22], the encircling steel wire in the SVW technique was improved to bone-penetrating tunnel steel wire, and a finite element analysis and clinical study were performed; the results suggested that this modified SVW (MSVW) technique has good biomechanical properties. However, the finite element analysis did not consider how the position of the transverse bone tunnel affected the internal fixation strength. Additionally, in the clinical study, some patients experienced complications such as steel wire loosening [22].

**Fig. 1:**
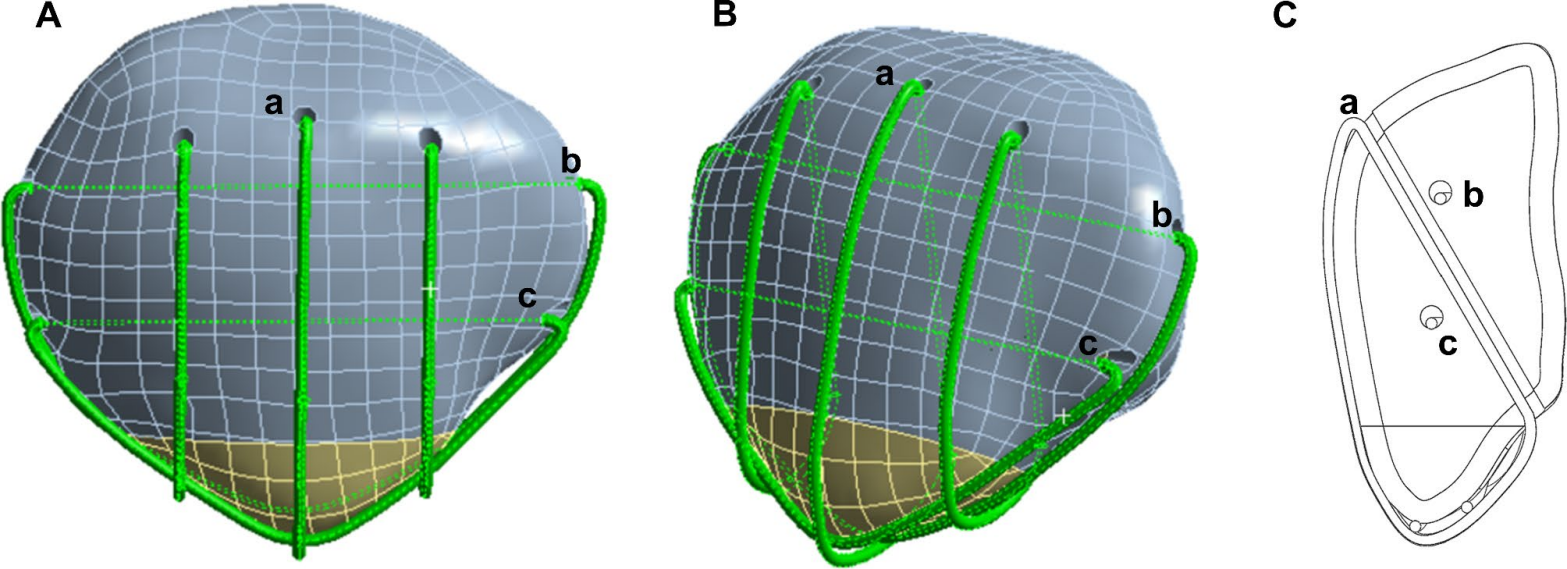
Candy box technical schematic diagram. (**A**) Front view of candy box technology. (**B**) Oblique view of candy box technology. (**C**) Side view of candy box technology. (**a**) Three discontinuous vertical steel wires. (**b**) Upper 1/3 steel wires of the patellar tunnel. (**c**) Middle 1/3 steel wires of the patellar tunnel

In summary, for inferior pole patellar fractures, unresolved issues remain regarding how to minimize complications while ensuring sufficient internal fixation strength and enabling early functional exercise of the knee joint. The purpose of this study was to address these issues. Considering the aforementioned situation, we attempted to further improve the MSVW technique by using intermittent vertical steel wire fixation combined with double cerclage through the proximal patella wire. The distribution of the improved steel wire is similar to that of a candy box; therefore, it is called the candy box (CB) technique. The main points of improvement were as follows: after fixing the broken end of the fracture with three interrupted vertical wires, lateral bone tunnels were established in the upper 1/3 and middle 1/3 of the patella; and the fixed wire around the periphery of the patella was replaced with two wires on the side of the patella through the bone tunnel (Fig. 1). This technique serves three purposes. First, it provides greater internal fixation strength by wrapping the fracture fragments of the lower pole of the patella like a CB. This helps prevent risks such as re-displacement of small fracture fragments. Second, it reduces detachment of the quadriceps tendon and minimizes complications such as soft tissue irritation. As a result, patients experience less pain during functional exercises. Third, this method ensures secure fixation and sufficient soft tissue protection, allowing patients to engage in early functional exercises for faster and better recovery.

**Fig. 2:**
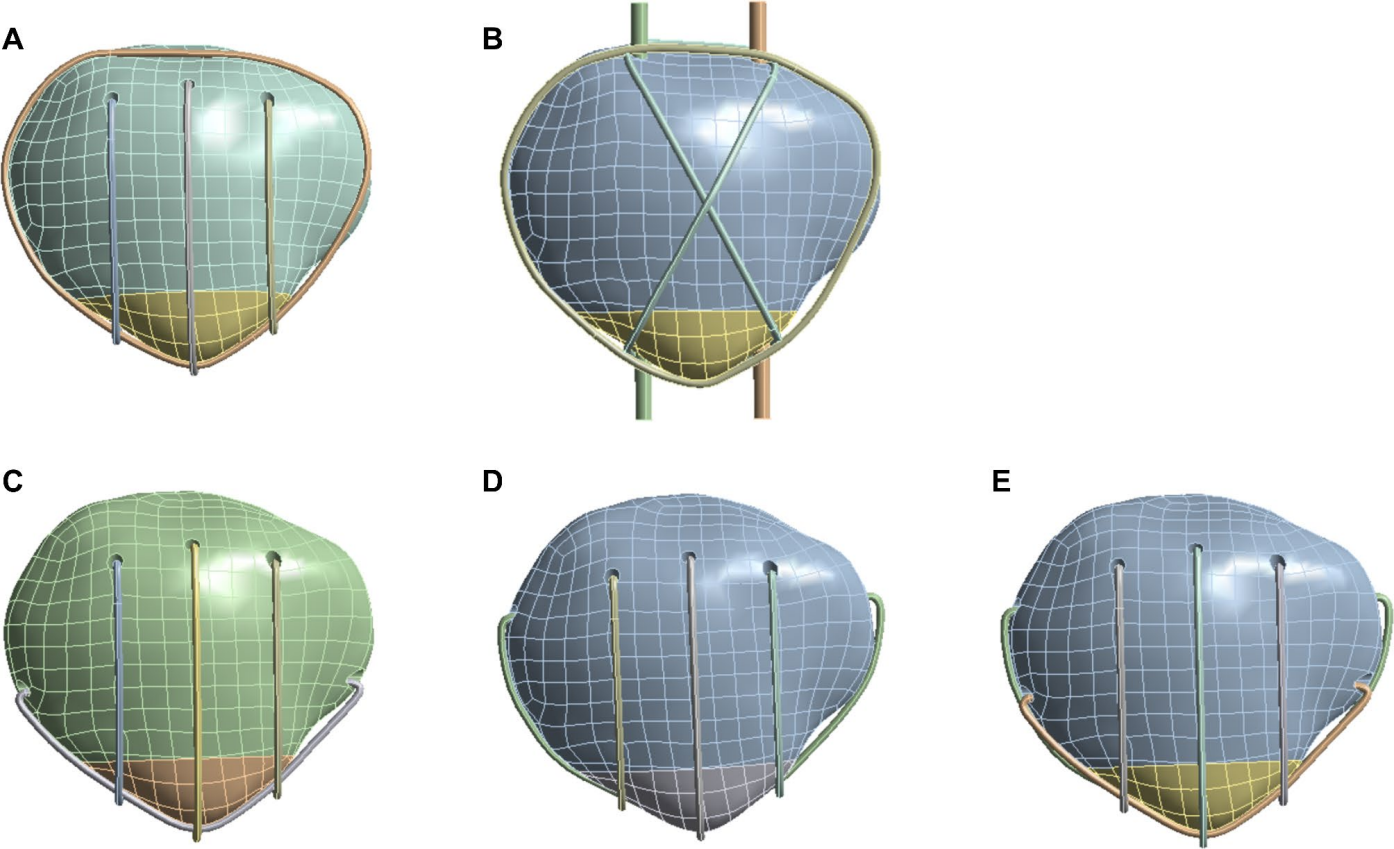
Diagram of different wiring techniques. (**A**) Separate vertical wiring combined with cerclage wiring (SVWC). (**B**) Tension-band wiring combined with cerclage wiring (TBWC). (**C**) Modified SVW with the middle 1/3 of the steel wire reserved (MSVW-A). (**D**) Modified SVW with the upper 1/3 of the steel wire reserved (MSVW-B). (**E**) Candy box technology (CB)

This study utilized finite element analysis and biomechanical experiments to compare the biomechanical characteristics of the CB technique with previously published techniques for treating inferior pole patellar fractures, including the conventional TBWC, emerging SVWC, and two versions of MSVW (Fig. 2A-E). Our hypothesis was that the CB technique would exhibit superior biomechanical properties.

## Materials and methods

### Establishment of the initial three-dimensional (3D) models

A 128-slice spiral computed tomography scanner (SIMENS SENSATION 128, slice thickness: 0.6 mm, slice gap: 0.6 mm, resolution: 512*512 pixels) was used to perform thin-layer scanning from the upper leg to the lower leg of an adult male volunteer laying on his back with his knee joint in a neutral position. The obtained two-dimensional image data were stored in DICOM format and imported into the 3D image modelling software processing system, Mimics21. The patella region was selected through region-growing, the patella polylines were extracted, and 3D models of the patella and inferior pole patellar fracture were established. The models were then input into Geomagic Studio 2021 software for noise removal, encapsulation, and smoothing, and the software’s surface modelling function was used to create a volume mesh solid.

### Establishment of the fracture model and evaluated internal fixation models

**Fig. 3:**
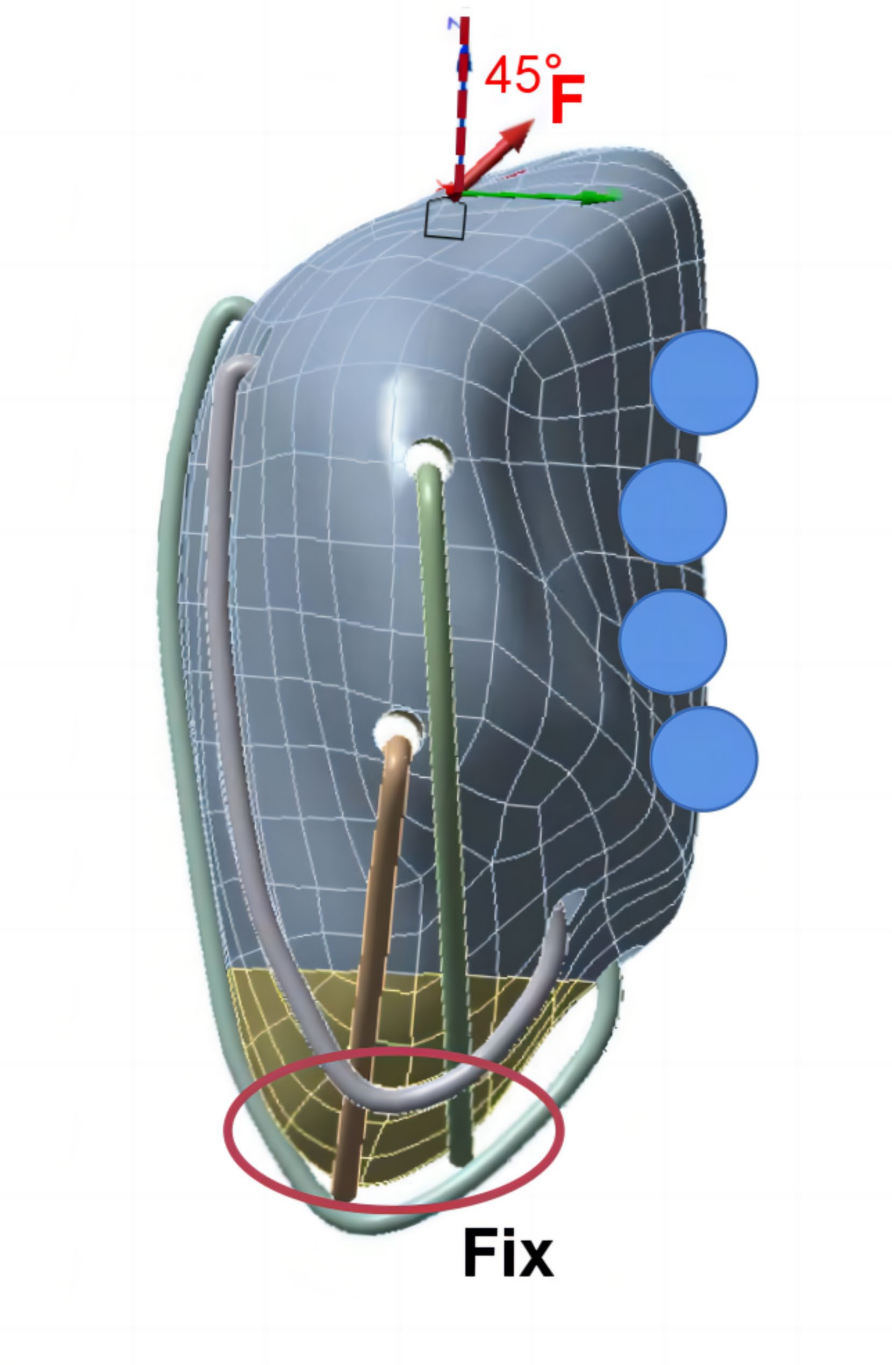
Schematic diagram of patella loading. The inferior pole of the patella is bound and constrained, the upper pole is loaded, and the arrow indicates the stretching direction. The blue circles represent the support of the medial and lateral condyles of the femur to the patella

The established 3D model of the patella fracture was used as input to the finite element analysis software, ANSYS WORKBENCH2021. Various parameters of the bone and wire in the 3D model were defined, such as the elastic modulus and Poisson’s ratio (as shown in Table 1) [4]. Three types of frictional contacts were set: the contact between the periphery of the patella and encircling wire, contact between the patella and vertical wire with a friction coefficient of 0.2 [23], and contact between fracture ends with a friction coefficient of 0.45 [24, 25]. The portion where the wire passed through the patella was set as “bonded” (i.e., there was no relative displacement between them). Five different techniques were used to construct five 3D models. We constrained the lower pole of the patella and applied loads on its upper pole. ‘Compression Only Support’ software was used behind the patella to simulate the medial and lateral condyles of femur, and an angle of 45° with respect to the long axis of patella was used to simulate forces on the knee joint [26] (Fig. 3).

**Table 1:** Model material parameters

Name of the material	Elastic modulus (MPa)	Poisson ratio
Cortical bone	10,000	0.30
Cancellous bone	840	0.29
Steel wire	100,000	0.29
Kirschner wire	200,000	0.30

### Evaluation indices

The solution module of ANSYS WORKBENCH 2021 software was used to analyse the stress and displacement distribution of the patella and internal fixation in each model under 100-N, 200-N, 300-N, 400-N, and 500-N loads.

### Biomechanical experiments

**Fig. 4:**
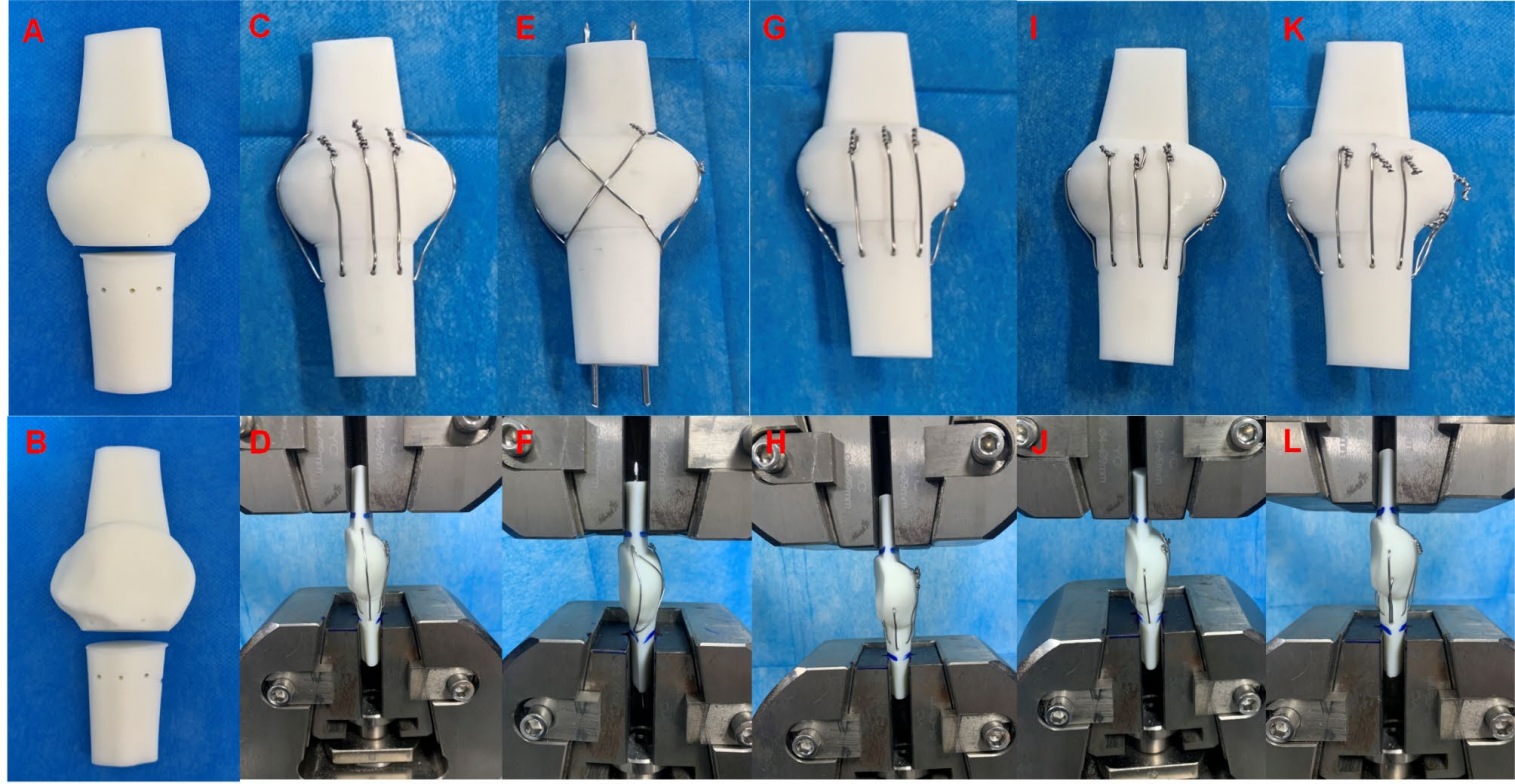
Construction of physical models and biomechanical experiments. (**A**) Front view of fracture model. (**B**) Back view of fracture model. (**C**-**D**) Separate vertical wiring combined with cerclage wiring (SVWC). (**E**-**F**) Tension-band wiring combined with cerclage wiring (TBWC). (**G**-**H**) Modified SVW with the middle 1/3 of the steel wire reserved (MSVW-A). (**I**-**J**) Modified SVW with the upper 1/3 of the steel wire reserved (MSVW-B). (**K**-**L**) Candy box technology (CB)

Due to its excellent comprehensive performance, acrylonitrile butadiene styrene (ABS) resin is widely used in 3D printing technology and biomechanical experiments [27, 28]. Based on the finite element model, we constructed solid fracture models of the above five internal fixation models with 3D printing using ABS photosensitive resin as the material [29–31], with each group consisting of five models (Fig. 4). One experienced physician was responsible for model preparation and ensuring accurate fixation of the metal wires. We applied constraints to the lower end of the patella and loaded force on its upper end at a set stretching speed of 1 mm/min, recording displacement data every 0.3 s. The tension was stopped when it reached 500 N. With ABS photosensitive resin as the material, there was no relative displacement inside the resin during stretching. The overall displacement of the upper and lower poles could only be caused by the displacement of the broken end. This allowed the electronic universal testing machine (Changzhou Sanfeng Instrument Technology Co., Ltd., China. Model number: MIT-100) to automatically record the overall displacement of the near fracture end using specific sensors, which was considered as the displacement of the fracture end. We then calculated the average displacement for each type of internal fixation under this load force (500 N).

### Statistical analysis

Statistical analyses were performed using SPSS 25.0 and Prism 9.0 software. Continuous variables are represented as the mean ± standard deviation. One-way analysis of variance (ANOVA) was used to compare displacement values between techniques under the same load, and the least squares difference t-test was used for multiple comparisons between techniques. The significance level was set at P < 0.05.

## Results

In the finite element analysis, the maximum displacement and maximum stress of the patella and steel wire under various force loads were lower with CB technology than with other techniques. In the biomechanical experiments, under a force of 500 N, the CB group exhibited the smallest average displacement (P < 0.05).

### Finite element analysis: displacement

**Fig. 5:**
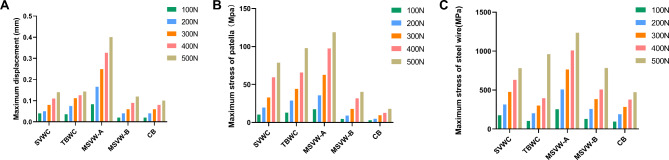
Histogram of displacement or stress at different forces. (**A**) The maximum displacement of different groups under different forces. (**B**) The maximum stress of patella under different forces. (**C**) The maximum stress of steel wire under different forces. Separate vertical wiring combined with cerclage wiring (SVWC). Tension-band wiring combined with cerclage wiring (TBWC). Modified SVW with the middle 1/3 of the steel wire reserved (MSVW-A). Modified SVW with the upper 1/3 of the steel wire reserved (MSVW-B). Candy box technology (CB)

We calculated and compared the displacement values for five different models; the results demonstrated that CB technology has lower displacement than that with other technologies under various force loads (See Fig. 5A, Additional file 1, Additional file 2).

**Fig. 6:**
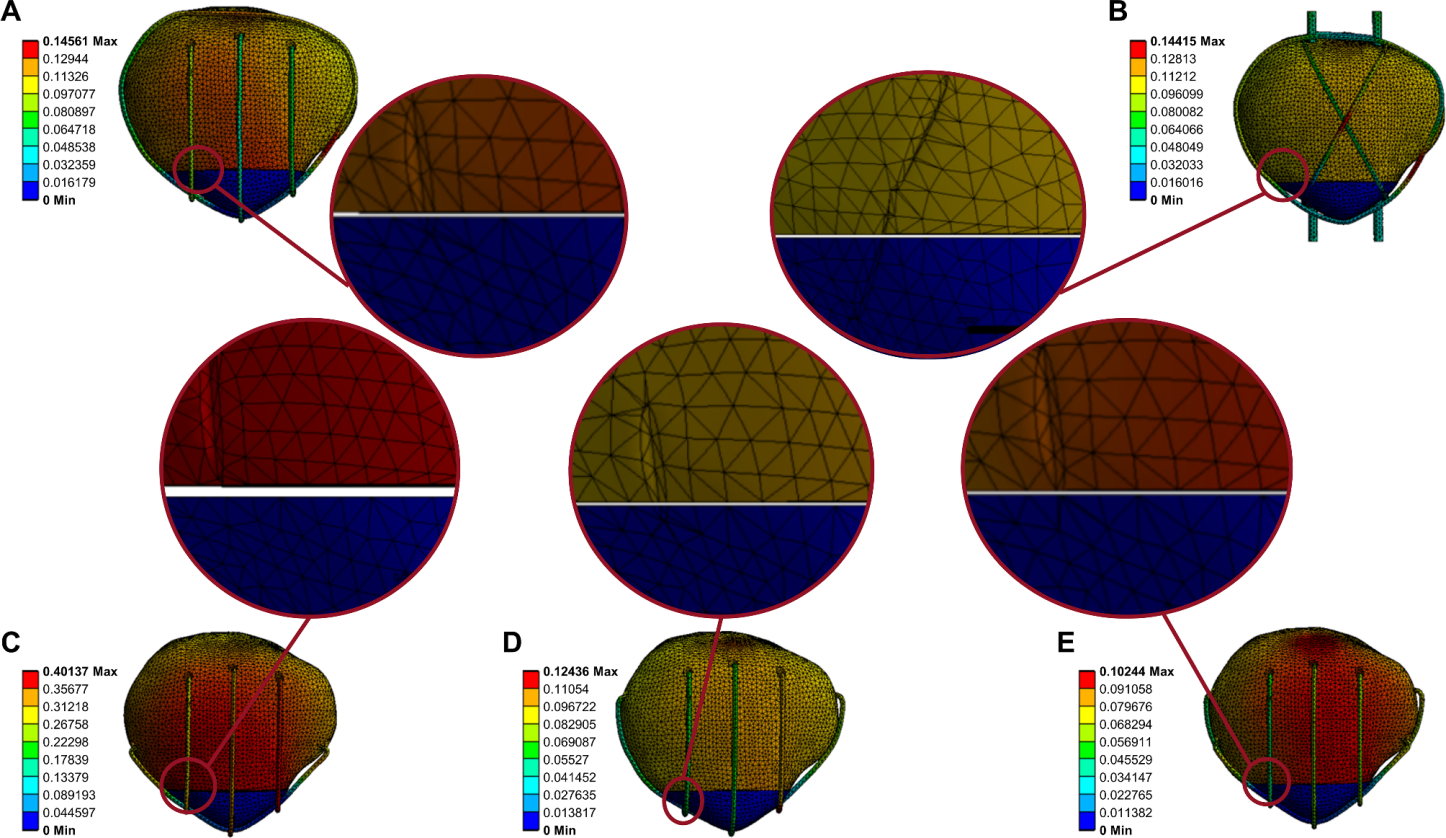
Displacement nephogram of different internal fixation groups under 500-N force. (**A**) Separate vertical wiring combined with cerclage wiring (SVWC). (**B**) Tension-band wiring combined with cerclage wiring (TBWC). (**C**) Modified SVW with the middle 1/3 of the steel wire reserved (MSVW-A). (**D**) Modified SVW with the upper 1/3 of the steel wire reserved (MSVW-B). (**E**) Candy box technology (CB)

The displacement nephogram under a 500-N load is shown in Fig. 6. Compared to that with the TBWC and SVWC techniques, the maximum displacement with the CB technique under a 500-N load was lower by 29% (0.144 mm vs. 0.102 mm) and 30% (0.145 mm vs. 0.102 mm), respectively. Compared to that with the MSVW-A and MSVW-B techniques, the maximum displacement with the CB technique under a 500-N load was lower by 75% (0.401 mm vs. 0.102 mm) and 18% (0.124 mm vs. 0.102 mm), respectively.

### Finite element analysis: stress

**Fig. 7:**
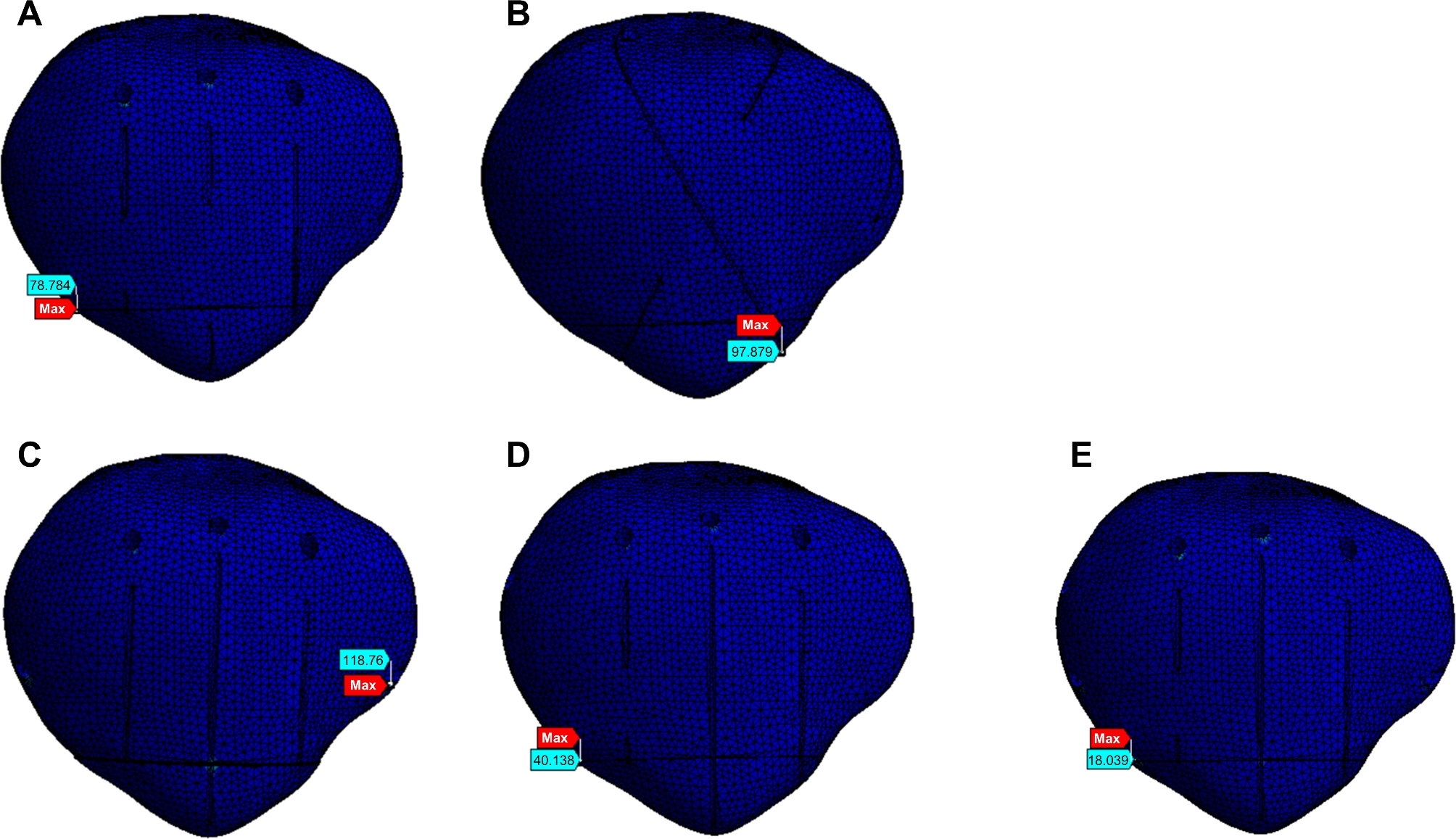
Stress nephogram of patella under 500-N force. (**A**) Separate vertical wiring combined with cerclage wiring (SVWC). (**B**) Tension-band wiring combined with cerclage wiring (TBWC). (**C**) Modified SVW with the middle 1/3 of the steel wire reserved (MSVW-A). (**D**) Modified SVW with the upper 1/3 of the steel wire reserved (MSVW-B). (**E**) Candy box Technology (CB)

**Fig. 8:**
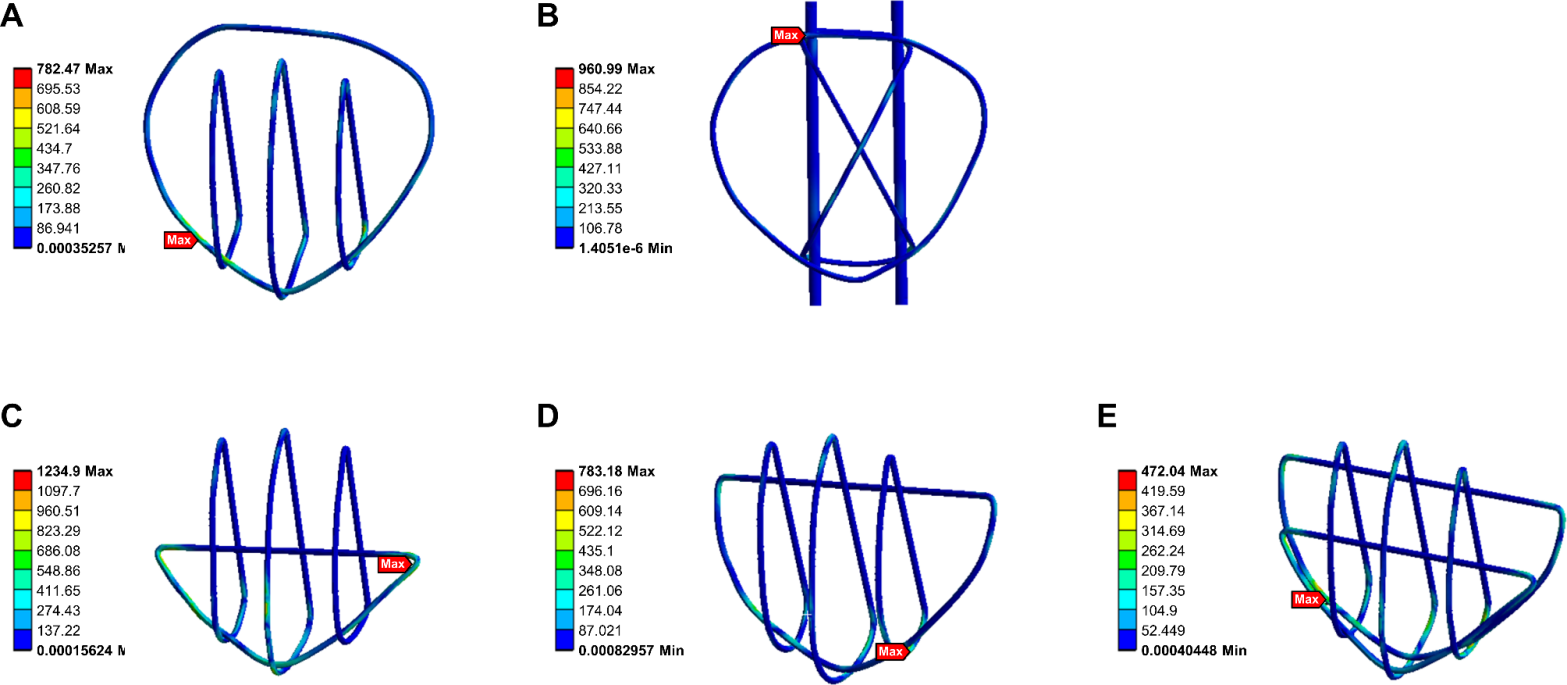
Stress nephogram of steel wire under 500-N force. (**A**) Separate vertical wiring combined with cerclage wiring (SVWC). (**B**) Tension-band wiring combined with cerclage wiring (TBWC). (**C**) Modified SVW with the middle 1/3 of the steel wire reserved (MSVW-A). (**D**) Modified SVW with the upper 1/3 of the steel wire reserved (MSVW-B). (**E**) Candy box technology (CB)

The CB technique exhibited the lowest maximum stress under different loads. The maximum stresses according to technique and load are shown in Fig. 5B for the patella and Fig. 5C for the steel wire. Stress nephograms under a 500-N load are shown in Fig. 7 (patella) and Fig. 8 (steel wire). Compared to that with the TBWC and SVWC techniques, the maximum stress of the patella with the CB technique under a 500-N load was lower by 81% (98 MPa vs. 18 MPa) and 77% (79 MPa vs. 18 MPa), respectively, while the maximum stress of the steel wire was lower by 51% (961 MPa vs. 472 MPa) and 40% (782 MPa vs. 472 MPa), respectively. Compared to that with the MSVW-A and MSVW-B technique, the maximum stress of the patella with the CB technique under a 500-N load was lower by 84% (118 MPa vs. 18 MPa) and 55% (40 MPa vs. 18 MPa), respectively, while the maximum stress of the steel wire was lower by 61% (1234 MPa vs. 472 MPa) and 39% (783 MPa vs. 472 MPa), respectively.

### Biomechanical experiments

**Fig. 9:**
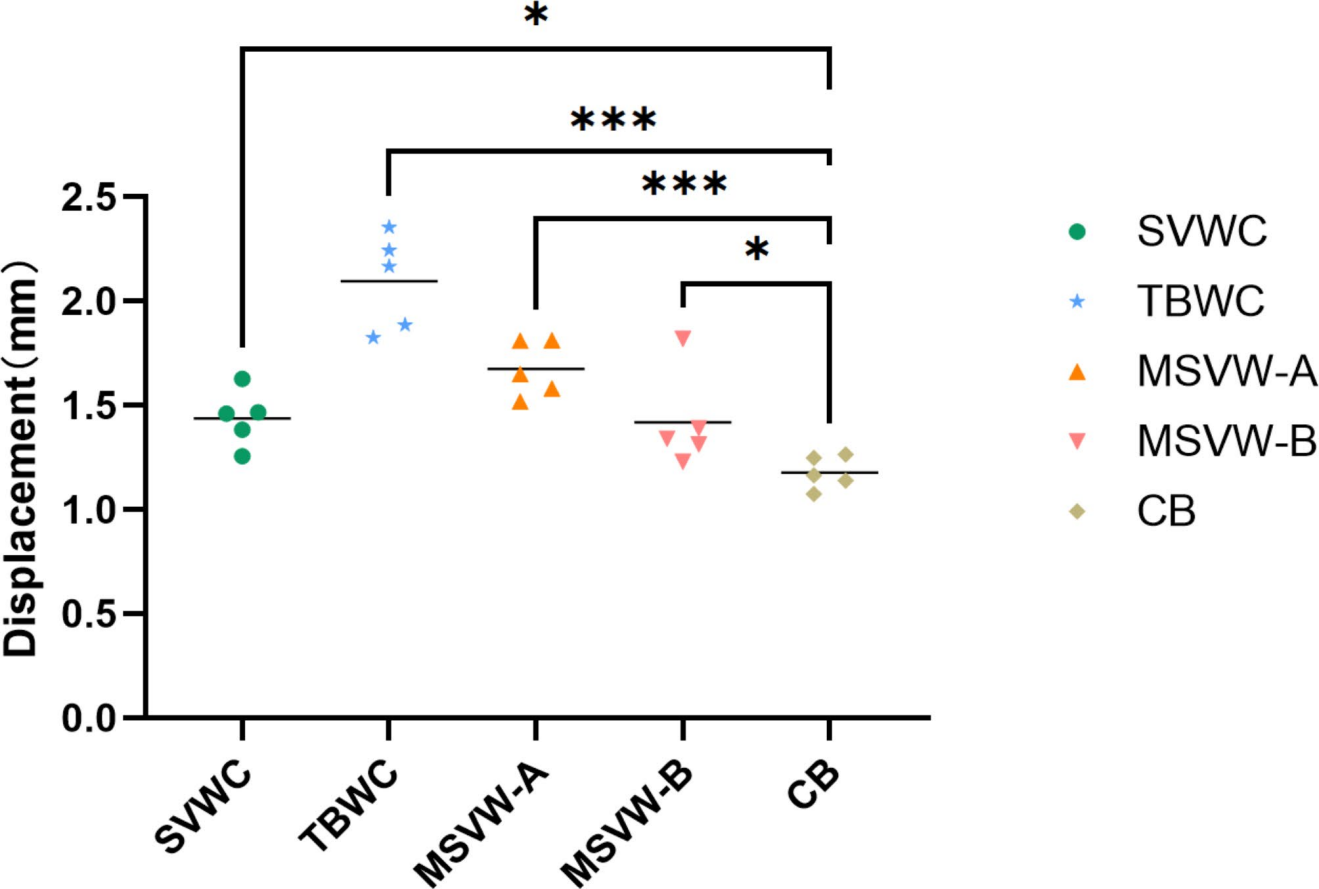
Displacement diagram of different internal fixation groups under 500-N force. Separate vertical wiring combined with cerclage wiring (SVWC). Tension-band wiring combined with cerclage wiring (TBWC). Modified SVW with the middle 1/3 of the steel wire reserved (MSVW-A). Modified SVW with the upper 1/3 of the steel wire reserved (MSVW-B). Candy box technology (CB). *p < 0.05; ***p < 0.001

The results of the biomechanical experiments demonstrated that, under a force of 500 N, the CB group exhibited the smallest average displacement (P < 0.05). Specifically, compared to that with the TBWC and SVWC techniques, the average displacement in the CB group was reduced by 43% (2.096 mm vs. 1.179 mm) and 18% (1.439 mm vs. 1.179 mm), respectively. Compared to that with the MSVW-A and MSVW-B techniques, the average displacement in the CB group was reduced by 27% (1.617 mm vs. 1.179 mm) and 17% (1.419 mm vs. 1.179 mm), respectively (Fig. 9).

## Discussion

Inferior pole patellar fractures are mostly comminuted fractures. Due to their small size and concentrated stress, it is difficult to maintain effective reduction and strong fixation, which makes their treatment challenging [4]. The methods reported in the literature have advantages and disadvantages, as well as various complications [1]. How to minimize complications while ensuring sufficient internal fixation strength and simultaneously meeting the early functional exercise needs of the knee joint remains an unresolved issue.

Among surgical methods for preserving the patella, the TBWC technique is considered as the most classic method for treating patellar fractures [32]. However, when it is used for inferior pole patellar fractures, owing to the crushing of the fracture block, the effect of fixation is relatively small when a steel needle is inserted into the inferior pole crushing site. Under early functional exercise, the risk of internal fixation failure and fracture non-union is still difficult to avoid [33]. Basket plate technology may limit knee joint movement in patients after surgery due to excessive stimulation of soft tissue and the potential risk of patellar tendon injury. [15, 16]. Although the patellar claw technique has a good fixation effect on transverse fractures of the patella, its excessive restorative force may cause small bone fragments to reposition in cases of inferior pole fractures. Additionally, detachment of the quadriceps tendon may exacerbate soft tissue injuries. [18].

SVWC technology has been specifically proposed for inferior pole patellar fractures. Although SVWC technology can concentrate the force of internal fixation on the inferior pole of the patella, the fixation strength is still limited, and the time and range of motion of the knee joint still need to be limited postoperatively; in addition, there are still a risk of postoperative complications, such as a slight limitation in knee extension and internal fixation fracture [34]. The MSVW technique as reported by He et al. in 2018 improved the encircling steel wire to a patellar tunnel steel wire based on the SVW technique [22]. However, it remained unclear whether one bone tunnel steel wire could satisfy the internal fixation strength requirements and whether different positions of the bone tunnel steel wire impacted the internal fixation strength. Furthermore, clinical studies revealed that some patients experienced complications with MSVW, specifically wire loosening.

Therefore, in order to solve the problem of how to minimize complications while ensuring sufficient internal fixation strength and meeting the early functional exercise requirements of the knee joint, we attempted to further improve the MSVW technique by using intermittent vertical steel wire fixation combined with double cerclage through the proximal patella wire. After fixing the fracture ends with three vertical steel wires, we created lateral bone tunnels in the upper and middle 1/3 of the patella. Then, we replaced the fixed steel wire around the patella with two steel wires on the side of the patella. This is the CB technology introduced in this article.

In order to verify the biomechanical stability of CB technology, we compared its biomechanical characteristics with previously proposed techniques (i.e. TBWC, SVWC, and MSVW) under different loads using finite element analysis and biomechanical experiments. The results indicate that compared to other techniques, CB technology has smaller displacements, wire stresses, and bone stresses under different loads. Further, the results of the comparison between MSVW-A and MSVW-B techniques indicated that the MSVW technique with only the middle 1/3 of steel wire reserved cannot obtain satisfactory internal fixation strength. This provides an answer to the debate about the placement of the bone tunnel steel wire in the SVW technique; specifically, the tunnel through the patella should be as close as possible to the upper 1/3 of the patella to obtain more satisfactory internal fixation strength.

Due to the improvement of one encircling steel wire to two bone tunnel steel wires, in addition to shortening the force arm, we added a bone tunnel steel wire to disperse stress, thereby improving the fixation strength. This may be one of the reasons why CB technology exhibits good biomechanical properties. Compared to the TBWC technique, the CB technique does not involve the insertion of Kirschner wires into the fractured bone fragment below the patella, which prevents further displacement of small fracture fragments and avoids irritation to soft tissues caused by longitudinal Kirschner wires. Compared to the basket-shaped steel plate and patellar claw techniques, the CB technique uses transverse bone tunnel steel wire for fixation. This eliminates the need to pass through the quadriceps tendon above the patella during surgery. Consequently, this procedure can be done with a smaller incision and without stripping the quadriceps tendon or affecting the blood supply above the patella. This eliminates the limitation of not being able to engage in early functional exercise due to excessive soft tissue stimulation. Compared to the MSVW technique, an additional bone tunnel steel wire was added based on existing technology. This provides stronger biomechanical stability, aiding in the avoidance of complications such as fracture displacement due to steel wire loosening during early functional exercise. More importantly, CB technology achieves, for the first time and in a true sense, the concentration of internal fixation stress on the lower pole of the patella by wrapping the fractured fragments like a ‘candy box’. This is unified with the biomechanical mechanism of the fracture itself, shifting the theoretical bias of internal fixation for lower pole patellar fractures towards central fixation at the lower pole and addressing current knowledge gaps. We consider this to be the most important reason for the excellent biomechanical characteristics of CB technology.

Because the internal fixation method in the CB technique goes through the patellar tunnel, it is noteworthy that a 2018 cadaver study showed that when penetrating the bone tunnel, destruction of the anterior cortex increases the risk of patellar fracture [35]. This was subsequently confirmed in a finite element analysis of the patellar tunnel [36]. In addition, a finite element analysis of the insertion position of the Kirschner wire showed that the more backward the Kirschner wire, the more stable the fracture [37]. Therefore, during the implementation of the CB technique, we attempted to maintain the position of the bone tunnel as far away from the anterior cortex as possible to minimise the risk of refracture due to the bone tunnel position. The stress nephograms of the steel wire and patella showed that the maximum stress with the CB technique appeared on the right side of the fracture end, and the stress of the two steel wires crossing the bone tunnel was not high. Therefore, we consider this method of crossing the bone tunnel as relatively safe and without an increased risk of refracture.

It is worth noting that the maximum stress of the steel wire in the finite element analysis structure reached 1234 MPa (MSVW-A), and the maximum stress of the patella reached 118 MPa (MSVW-A). This may be due to the phenomenon of ‘stress shielding’, which has been widely documented in studies of bone implant interactions [38]. When components with different elastic moduli bear loads in parallel, components with higher elastic moduli bear more of the load, providing stress and strain shielding for components with lower elastic moduli. This leads to much higher stress in the steel wire than in the patella at the same location, which may ultimately lead to complications such as bone loss and stress shielding osteoporosis [39, 40], This indirectly proves that MSVW technology, which only retains the middle 1/3 of the steel wire, cannot achieve sufficient stability. Furthermore, based on national standards for medical steel wires, 1234 MPa does not reach the yield strength of the steel wire; additionally, research has shown that 118 MPa is far from reaching the yield strength of the patella bone [41]. Our subsequent biomechanical experiments also confirmed this point, as there was no occurrence of internal fixation failure under a force of 500 N.

Previous studies have shown that the average maximum tension that can be applied by the quadriceps tendon during knee extension is 316 N. In addition, a previous biomechanical experiment reported that the maximum load at which a repaired patellar tendon fails is 433 N [42]. Furthermore, studies have indicated that when the patella is subjected to a force inclined upward at an angle of 45°, the pressure on the patellofemoral joint reaches its maximum value [43, 44]. Encouragingly, our finite element analysis results indicate that CB technology can achieve minimal displacement and stress under different force loadings at 45°. Additionally, the biomechanical experimental results showed that the CB group still had the smallest displacement when the load reached 500 N. Because CB technology can avoid the irritation of longitudinal Kirschner wires on soft tissues caused by TBWC, it can successfully break the limitation against early exercise to protect the quadriceps tendon. The additional steel wire compared to that with the MSVW technique can make the lower pole of patella more inclined towards ‘central fixation’. In addition, our research shows that under experimental conditions in which the patella is subjected to the maximum load, CB technology still has sufficiently high internal fixation strength. Therefore, CB technology should be considered as safe for similar postoperative treatments. Further, based on the present findings, we preliminarily consider that the need for early functional exercise in patients with inferior pole patellar fractures can be met with the application of CB technology in clinical practice, while maintaining satisfactory internal fixation strength and reducing complications.

There are several limitations to this study. Firstly, the modelling of the knee joint and its internal fixation requires complex details and constraints [45], and our model could not accurately simulate the motion process of the knee joint. Secondly, the finite element model did not consider material anisotropy and lacked an analysis of different angles and types of fractures. Moreover, the impact of patellar nerve traction and the possible biomechanical changes that may occur during fracture healing were not considered [46, 47]. Finally, clinical data to validate the results of this research were lacking. Therefore, later research stages must establish models from different perspectives, forces, and types of fractures to verify the biomechanical performance of CB technology. In addition, finite element and biomechanical models should be further improved by considering material anisotropy, establishing fracture healing models, and using fresh cadaveric specimens to simulate biomechanical processes under realistic conditions. Lastly, long-term clinical studies are needed to validate the treatment efficacy of CB technology for inferior pole patellar fractures.

## Conclusion

In summary, compared to other internal fixation methods evaluated in this study, the CB technique shows smaller displacement and stress, providing a more secure fixation. However, the establishment of additional models is still required to verify its biomechanical performance under different angles, forces, and fracture types. Clinical trials are also needed to evaluate its therapeutic effects. We look forward to further exploring CB technology as a fixed and reliable internal fixation method, with fewer complications, that can meet the early functional exercise needs of the knee joint.

### Electronic supplementary material

Below is the link to the electronic supplementary material.

Additional file 1: Supplementary Table S1: The mean displacement of the fracture ends

Additional file 2: Supplementary Figure 1: Histogram of the mean displacement of the fracture ends. Separate vertical wiring combined with cerclage wiring (SVWC). Tension-band wiring combined with cerclage wiring (TBWC). Modified SVW with the middle 1/3 of the steel wire reserved (MSVW-A). Modified SVW with the upper 1/3 of the steel wire reserved (MSVW-B). Candy box technology (CB)

## Data Availability

The data used and/or analysed during the current study are available from the corresponding author upon reasonable request.

## Abbreviations

CB: Candy box
SVWC: Separate vertical wiring combined with cerclage wiring
TBWC: Tension-band wiring combined with cerclage wiring
MSVW-A: Modified SVW with the middle 1/3 of the steel wire reserved
MSVW-B: Modified SVW with the upper 1/3 of the wire reserved
3D: Three-dimensional

## References

[1] Pu S, Chen Y, Liang J, Xu Y, Zhao Y (2022). Treatment of inferior Pole fracture of the patella with tension-free external immobilization. BMC Surg.

[2] Zhou M, Jia X, Cao Z, Ma Y, Wang Y, Wang P (2023). Treatment of inferior Pole patella fracture using Krackow suturing combined with the suture bridge technique. Arch Orthop Trauma Surg.

[3] Heusinkveld MH, den Hamer A, Traa WA, Oomen PJ, Maffulli N (2013). Treatment of transverse patellar fractures: a comparison between metallic and non-metallic implants. Br Med Bull.

[4] Du B, Ma T, Bai H, Lu Y, Xu Y, Yang Y (2022). Efficacy comparison of Kirschner-Wire tension band combined with patellar cerclage and anchor-loop plate in treatment of inferior patellar Pole fracture. Front Bioeng Biotechnol.

[5] Matejcić A, Puljiz Z, Elabjer E, Bekavac-Beslin M, Ledinsky M (2008). Multifragment fracture of the patellar apex: basket plate osteosynthesis compared with partial patellectomy. Arch Orthop Trauma Surg.

[6] Chang CH, Chuang HC, Su WR, Kuan FC, Hong CK, Hsu KL (2021). Fracture of the inferior Pole of the patella: tension band wiring versus transosseous reattachment. J Orthop Surg Res.

[7] Oh HK, Choo SK, Kim JW, Lee M (2015). Internal fixation of displaced inferior Pole of the patella fractures using vertical wiring augmented with krachow suturing. Injury.

[8] Fan M, Wang D, Sun K, Jiang W (2020). Study of double button plate fixation in treatment of inferior Pole of patella fracture. Injury.

[9] Ma XY, Cui D, Liu B, Wang Z, Yu HL, Yuan H (2023). Treating inferior Pole fracture of patella with hand plating system: first clinical results. Orthop Surg.

[10] Kim KS, Suh DW, Park SE, Ji JH, Han YH, Kim JH (2021). Suture anchor fixation of comminuted inferior Pole patella fracture-novel technique: suture bridge anchor fixation technique. Arch Orthop Trauma Surg.

[11] Jang JH, Cho YJ, Choi YY, Rhee SJ (2021). Hammock plating for comminuted inferior sleeve avulsion fractures of the patella: a surgical technique and clinical results. Orthop Traumatol Surg Res.

[12] Bonnaig NS, Casstevens C, Archdeacon MT, Connelly C, Monaco N, Wyrick JD (2015). Fix it or discard it? A retrospective analysis of functional outcomes after surgically treated patella fractures comparing ORIF with partial patellectomy. J Orthop Trauma.

[13] Kakazu R, Archdeacon MT (2016). Surgical management of patellar fractures. Orthop Clin North Am.

[14] Zhu W, Xie K, Li X, Li L, Yang J, Xu L (2020). Combination of a miniplate with tension band wiring for inferior patellar Pole avulsion fractures. Injury.

[15] He QF, Pan GB, Yu ZF, Yao WX, Zhu LL, Luo CF (2021). Novel rim plating technique for treatment of the inferior Pole fracture of the patella. Orthop Surg.

[16] Veselko M, Kastelec M (2005). Inferior patellar Pole avulsion fractures: osteosynthesis compared with Pole resection. Surgical technique. J Bone Joint Surg Am.

[17] Zhang Y, Wang P, Xia Y, Zhou P, Xie Y, Xu S (2017). Application of a shape-memory alloy concentrator in displaced patella fractures: technique and long-term results. J Knee Surg.

[18] Chen R, Cao H, Sun Z, Jiang L, Li X, Zhao L (2022). The clinical outcome of the reduction of the patellar inferior Pole fracture with wire cerclage through a generated bone hole, in combination with patellar concentrator: a retrospective comparative study. J Orthop Surg Res.

[19] Fan W, Xiao Y, Xiang F, Yang Y (2022). Research progress of inferior Pole patellar fracture: a systematic review. Asian J Surg.

[20] Yang KH, Byun YS (2003). Separate vertical wiring for the fixation of comminuted fractures of the inferior Pole of the patella. J Bone Joint Surg Br.

[21] Song HK, Yoo JH, Byun YS, Yang KH (2014). Separate vertical wiring for the fixation of comminuted fractures of the inferior Pole of the patella. Yonsei Med J.

[22] He S, Huang X, Yan B, Zhu J, Bao N, Zhao J (2018). Modified technique of separate vertical wiring for the fixation of patellar inferior Pole fracture. J Orthop Trauma.

[23] Zhu W, Xu L, Xie K, Li X, Zhang X, Fang S (2022). Design and validation of a smile-necklace plate for treating inferior patellar Pole avulsion fractures: a review and hypothesis. Orthop Surg.

[24] Chang CW, Chen YN, Li CT, Chung YH, Chang CH, Peng YT (2018). Role of screw proximity in the fixation of transverse patellar fractures with screws and a wire. J Orthop Surg (Hong Kong).

[25] Demirtaş Y, Katı YA (2023). A novel patella fracture fixation technique: finite element analysis. Arch Orthop Trauma Surg.

[26] Chen CH, Chen YN, Li CT, Chang CW, Chang CH, Peng YT (2019). Roles of the screw types, proximity and anterior band wiring in the surgical fixation of transverse patellar fractures: a finite element investigation. BMC Musculoskelet Disord.

[27] Wallace M, Johnson DB, Pierce W, Iobst C, Riccio A, Wimberly RL (2019). Biomechanical assessment of torsional stiffness in a supracondylar humerus fracture model. J Pediatr Orthop.

[28] Fan L, Wei L, Zhu Y, Wang Y, Fei J, Li Y (2020). Synthesis of environmentally friendly acrylonitrile butadiene styrene resin with low VOC. Mater (Basel).

[29] Arciero RA, Parrino A, Bernhardson AS, Diaz-Doran V, Obopilwe E, Cote MP (2015). The effect of a combined glenoid and Hill-Sachs defect on glenohumeral stability: a biomechanical cadaveric study using 3-dimensional modeling of 142 patients. Am J Sports Med.

[30] Reymus M, Fabritius R, Keßler A, Hickel R, Edelhoff D, Stawarczyk B (2020). Fracture load of 3D-printed fixed dental prostheses compared with milled and conventionally fabricated ones: the impact of resin material, build direction, post-curing, and artificial aging-an in vitro study. Clin Oral Investig.

[31] Wang YC, Yang D, Zhao LH, Xiao B, Ma QC, Dong LC (2023). Finite element analysis of mechanical characteristics of internal fixation for treatment of proximal femoral osteolytic lesions in children. Orthop Surg.

[32] Meng D, Meng Y, Li B, Zeng G, Zhang B, Hou C (2021). Comparison between tension band and cerclage with X-Plate and lag screws in treatment of comminuted patellar fractures. J Orthop Sci.

[33] Matthews B, Hazratwala K, Barroso-Rosa S (2017). Comminuted patella fracture in elderly patients: a systematic review and case report. Geriatr Orthop Surg Rehabil.

[34] Cho JW, Kim J, Cho WT, Gujjar PH, Oh CW, Oh JK (2018). Comminuted inferior Pole fracture of patella can be successfully treated with rim-plate-augmented separate vertical wiring. Arch Orthop Trauma Surg.

[35] Bonazza NA, Lewis GS, Lukosius EZ, Roush EP, Black KP, Dhawan A (2018). Effect of transosseous tunnels on patella fracture risk after medial patellofemoral ligament reconstruction: a cadaveric study. Arthroscopy.

[36] Wierer G, Winkler PW, Pomwenger W, Plachel F, Moroder P, Seitlinger G (2022). Transpatellar bone tunnels perforating the lateral or anterior cortex increase the risk of patellar fracture in MPFL reconstruction: a finite element analysis and survey of the International Patellofemoral Study Group. Knee Surg Sports Traumatol Arthrosc.

[37] Ling M, Zhan S, Jiang D, Hu H, Zhang C (2019). Where should Kirschner wires be placed when fixing patella fracture with modified tension-band wiring? A finite element analysis. J Orthop Surg Res.

[38] Amirouche F, Solitro GF, Walia A, Gonzalez M, Bobko A (2017). Segmental acetabular rim defects, bone loss, oversizing, and press fit cup in total hip arthroplasty evaluated with a probabilistic finite element analysis. Int Orthop.

[39] Pettersen SH, Wik TS, Skallerud B (2009). Subject specific finite element analysis of stress shielding around a cementless femoral stem. Clin Biomech (Bristol Avon).

[40] Garabano G, Rodriguez J, Perez Alamino L, Pesciallo CA, Del Sel H, Lopreite F (2022). Stress shielding in total knee replacements: comparative analysis between titanium and all-polyethylene bases at 10 years follow-up. J Orthop.

[41] Kerrigan JR, Sanchez-Molina D, Neggers J, Arregui-Dalmases C, Velazquez-Ameijide J, Crandall JR (2014). Indentation response of human patella with elastic modulus correlation to localized fractal dimension and bone mineral density. J Mech Behav Biomed Mater.

[42] Massey PA, Myers M, McClary K, Brown J, Barton RS, Solitro GF (2020). Biomechanical analysis of patellar tendon repair with knotless suture anchor tape versus transosseous suture. Orthop J Sports Med.

[43] Dargel J, Gick S, Mader K, Koebke J, Pennig D (2010). Biomechanical comparison of tension band- and interfragmentary screw fixation with a new implant in transverse patella fractures. Injury.

[44] Amis AA (2007). Current concepts on anatomy and biomechanics of patellar stability. Sports Med Arthrosc Rev.

[45] Amirouche F, Solitro GF (2011). Challenges in modeling total knee arthroplasty and total hip replacement. Procedia Iutam.

[46] Kerns J, Piponov H, Helder C, Amirouche F, Solitro G, Gonzalez M (2019). Mechanical properties of the human tibial and peroneal nerves following stretch with histological correlations. Anat Rec (Hoboken).

[47] Travascio F, Buller LT, Milne E, Latta L (2021). Mechanical performance and implications on bone healing of different screw configurations for plate fixation of diaphyseal tibia fractures: a computational study. Eur J Orthop Surg Traumatol.

