# Supplementary material for: Candy box technique for the fixation of inferior pole patellar fractures: finite element analysis and biomechanical experiments
Source: BMC Musculoskelet Disord. 2023 Oct 23;24:835. doi: 10.1186/s12891-023-06946-1 (PMC10594795; doi:10.1186/s12891-023-06946-1)
Supplement: Supplementary file 1 — Additional file 1: Supplementary Table S1: The mean displacement of the fracture ends [file 12891_2023_6946_MOESM1_ESM.docx]

**Additional file 1****: Supplementary Table S1: The mean displacement of the fracture ends.**

We collected displacement values at 10 points on the fracture ends in our finite element model and performed statistical analysis. The results indicated that, when subjected to various force loads, the CB technique exhibited the lowest average displacement at the fracture ends (P<0.05).

Supplementary Table S1: The mean displacement of the fracture ends.

| Force  Group | 100N | 200N | 300N | 400N | 500N |
| --- | --- | --- | --- | --- | --- |
|  | mean displacement（mm,ｘ±ｓ) | | | | |
| A(SVWC) | 0.0258±0.00372 | 0.0510±0.00267 | 0.0770±0.00403 | 0.1020±0.00567 | 0.1260±0.00714 |
| B(TBWC) | 0.0256±0.00094 | 0.0525±0.00183 | 0.0786±0.00295 | 0.0935±0.00348 | 0.1098±0.00411 |
| C(MSVW-A) | 0.0790±0.00274 | 0.1578±0.00547 | 0.2366±0.00820 | 0.3108±0.01054 | 0.3778±0.01347 |
| D(MSVW-B) | 0.0226±0.00139 | 0.0452±0.00276 | 0.0677±0.00422 | 0.0894±0.00546 | 0.0997±0.00556 |
| E(CB) | 0.0185±0.00138 | 0.0374±0.00274 | 0.0559±0.00434 | 0.0744±0.00567 | 0.0916±0.00789 |
| F value | 1210 | 2259 | 2185 | 2268 | 2168 |
| P value | ＜0.0001 | ＜0.0001 | ＜0.0001 | ＜0.0001 | ＜0.0001 |

Note: (A) separate vertical wiring combined with cerclage wiring (SVWC). (B)Tension-band wiring combined with cerclage wiring (TBWC). (C)Modified SVW with the middle 1/3 of the steel wire reserved (MSVW-A). (D)Modified SVW with the upper 1/3 of the steel wire reserved (MSVW-B). (E)candy box technology (CB). *P*<0.05 indicates that the difference is significant.
