# Supplementary figures and images for: Candy box technique for the fixation of inferior pole patellar fractures: finite element analysis and biomechanical experiments
Source: BMC Musculoskelet Disord. 2023 Oct 23;24:835. doi: 10.1186/s12891-023-06946-1 (PMC10594795; doi:10.1186/s12891-023-06946-1)

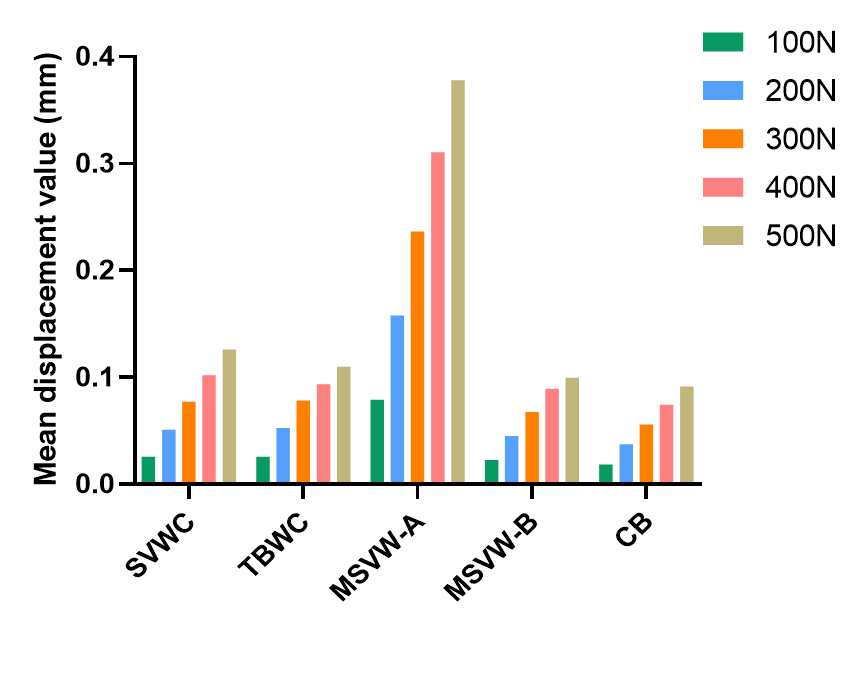

Supplement: Supplementary file 2 — Additional file 2: Supplementary Figure 1: Histogram of the mean displacement of the fracture ends. Separate vertical wiring combined with cerclage wiring (SVWC). Tension-band wiring combined with cerclage wiring (TBWC). Modified SVW with the middle 1/3 of the steel wire reserved (MSVW-A). Modified SVW with the upper 1/3 of the steel wire reserved (MSVW-B). Candy box technology (CB) [file 12891_2023_6946_MOESM2_ESM.png]
